# Birdlike Takeoff Strategies Enabled by Flapping–Pitch Effect in a Meter-Scale Flapping-Wing Robot

**DOI:** 10.34133/cbsystems.0714

**Published:** 2026-10-16

**Authors:** Haoyu Wang, Siping Zhong, Yihang Shi, Zhenkun Gong, Jizhou Jiang, Zihao Wei, Erzhen Pan, Junzhi Yu, Wenfu Xu

**Affiliations:** ^1^ School of Robotics and Advanced Manufacture, Harbin Institute of Technology, Shenzhen 518052, China.; ^2^ Guangdong Provincial Key Laboratory of Intelligent Morphing Mechanisms and Adaptive Robots, Shenzhen 518052, China.; ^3^ Guangdong Biomimetic Intelligent Unmanned System Engineering Technology Research Center, Shenzhen 518052, China.; ^4^ State Key Laboratory for Turbulence and Complex Systems, Department of Advanced Manufacturing and Robotics, College of Engineering, Peking University, Beijing 100871, China.

## Abstract

The takeoff strategy in birds involves a fundamental trade-off between acceleration and climb, yet its physical consequences are difficult to isolate in biological experiments. Here, we develop a meter-scale flapping-wing robot for controlled investigation of takeoff strategies. We show that a flapping–pitch effect, arising from flapping kinematics and aeroelastic interactions, enables pitch modulation at low speeds where conventional control surfaces have limited authority. In parallel, we establish a lattice-Boltzmann-method (LBM)-based 2-way fluid–structure interaction (FSI) framework for the full-process simulation of flapping-wing takeoff. To the best of our knowledge, this is the first LBM-based 2-way FSI analysis that resolves the complete takeoff dynamics of a meter-scale flapping-wing robotic system. Under the tested conditions, the 15° low-angle strategy increased the total flight distance by 49% relative to that of the 65° strategy and reached a comparable altitude at the 3-s evaluation time. These results provide engineering-based physical evidence that may help interpret certain avian takeoff strategies and highlight the role of the flapping–pitch effect in low-speed flight control.

## Introduction

Avoiding predation is a central challenge for animal survival and a major evolutionary driver shaping avian morphology and behavior, particularly takeoff and escape performance. From the prey’s perspective, escape success depends on both intrinsic factors and environmental conditions [1–4]. A general consensus is that escape aims to maximize distance from predators by exploiting either individual performance or environmental advantages [5–7]. Under fixed conditions, the choice of takeoff strategy may further influence escape effectiveness.

Flight involves continuous conversion between kinetic energy (speed) and potential energy (altitude) and is therefore strongly influenced by climb angle during takeoff [8,9]. Takeoff in birds involves a fundamental trade-off between acceleration and climb, which directly influences escape performance. Shallow takeoff angles favor rapid acceleration and horizontal distance gain, whereas steep angles enable faster altitude acquisition (Fig. 1) [6,10]. Although this trade-off has been widely observed, its optimality and underlying physical mechanisms remain unresolved.

**Fig. 1.**
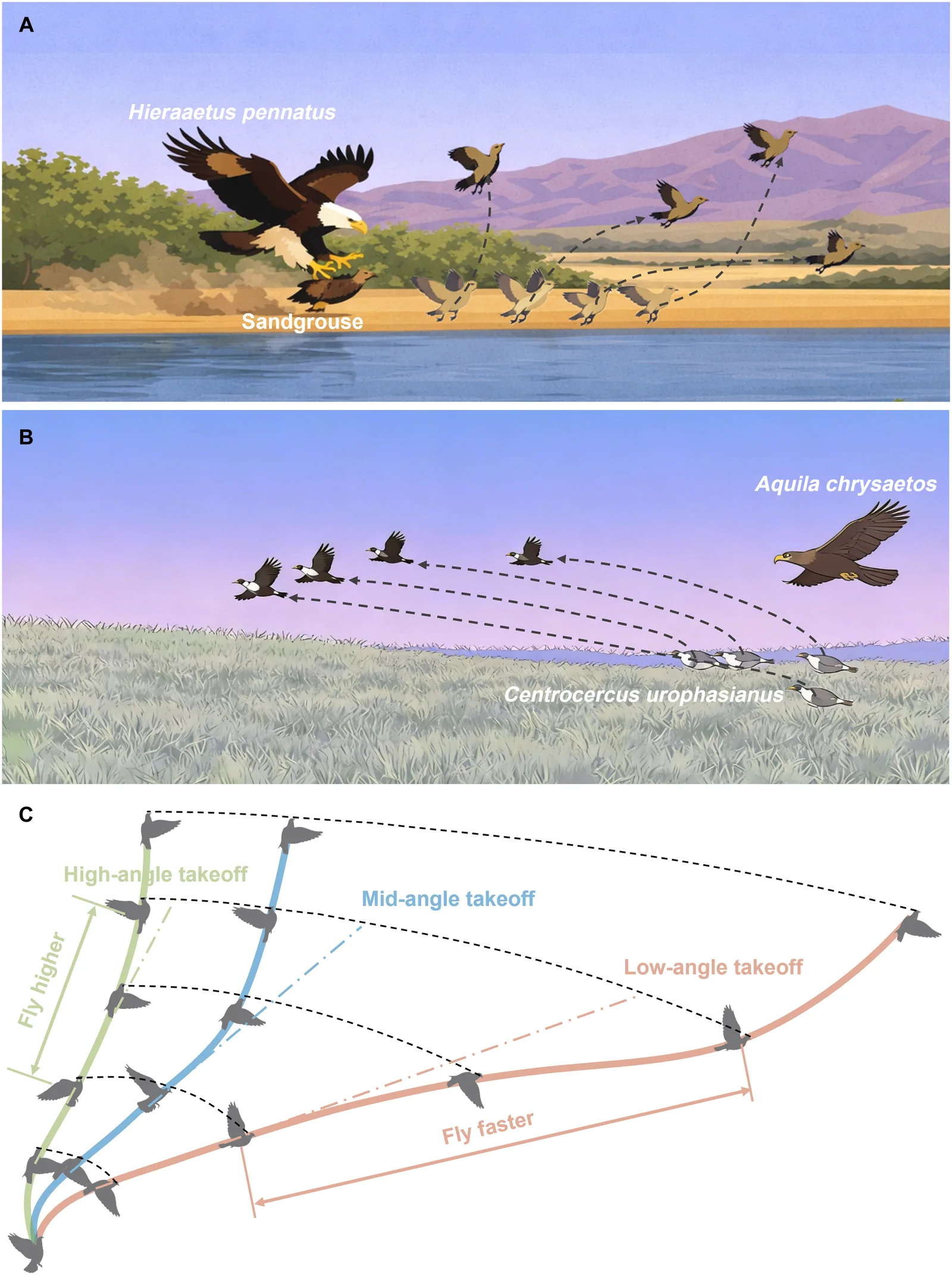
Takeoff strategies in birds. (A) Escape takeoff of a sandgrouse under predation by a *Hieraaetus pennatus* (Movie S2). This case illustrates diversified strategies within a species: some individuals adopt a low-angle, rapid-acceleration takeoff, whereas others choose high-angle, rapid-climb trajectories. Although the high-angle takeoff observed in the video results in predation—consistent with our hypothesis—we do not consider this outcome to be generalizable. Instances also exist where individuals adopting a low-angle acceleration are captured. Ultimately, capture outcomes depend on a broader set of factors, including environmental conditions and individual variability, and therefore cannot serve as direct evidence for the superiority of a given takeoff strategy. (B) Escape takeoff of *Centrocercus urophasianus* under predation by an *Aquila chrysaetos* (Movie S2). In contrast, this species exhibits a consistent strategy, with nearly all individuals adopting a low-angle, rapid-acceleration takeoff. (C) Schematic trajectories for different climb angles. High takeoff angles favor rapid altitude gain, whereas low angles favor faster acceleration.

A key challenge lies in the limited controllability and repeatability of biological experiments [2,11], which makes it difficult to isolate the effects of different takeoff strategies under identical conditions. Variations in environmental factors, individual conditions, and initial state further complicate quantitative comparison, preventing systematic evaluation of how takeoff angle influences flight performance.

To address these limitations, we develop a meter-scale flapping-wing robotic platform that enables controlled and repeatable investigation of takeoff strategies. The platform supports multiangle takeoff under consistent conditions, allowing direct comparison of acceleration, climb, and flight distance. Compared with existing flapping-wing systems [12–14], which are often constrained by limited thrust or reduced control authority at low speeds, this platform achieves sufficient thrust-to-weight ratio (TWR) and low-speed maneuverability for the systematic analysis of takeoff dynamics [15–17].

In this study, we reveal a flapping–pitch coupling effect arising from flapping kinematics and aeroelastic interactions, in which modulation of flapping motion redistributes aerodynamic forces and generates controllable pitching moments. This effect provides an effective mechanism for pitch control in low-speed regimes where conventional aerodynamic surfaces become ineffective. While similar phenomena have been qualitatively observed in biological flight [18–20], their functional role in takeoff strategy has not been quantitatively established.

Wing flexibility and the bidirectional interaction between structural deformation and the surrounding flow are central to flapping-wing aerodynamics. Hudson et al. [21] coupled a rigid multibody vehicle, including rigid wings and legs, with a modified strip-theory aerodynamic model. Hashimoto et al. [22] represented takeoff through the prescribed motion of rigid bodies in a one-way flow simulation. To capture these interactions throughout takeoff, we developed a lattice-Boltzmann-method (LBM)-based 2-way fluid–structure interaction (FSI) framework for the full-process analysis of a meter-scale flapping-wing robot. The method directly resolves aerodynamic-load-driven wing deformation and returns that deformation to the flow at every coupling step, while the resulting unsteady loads drive body motion and the complete takeoff trajectory. To our knowledge, this is the first application of an LBM-based 2-way FSI framework to full-process takeoff analysis of a meter-scale flapping-wing robot.

By combining controlled robotic experiments and coupled simulations, we show that the tested takeoff strategies produce distinct temporal allocations of kinetic and potential energy. During avian escape, the initial takeoff is crucial because split-second choices determine how rapidly the animal gains separation, speed, and climb margin [6,10]. We therefore use 3 s after release as a common engineering evaluation point. At this point, the tested strategies have completed the release transient, established sufficient speed and altitude for subsequent maneuvers, and reached comparable altitude-, speed-, and energy-related states; accumulated distance then provides a controlled measure of strategy-dependent performance. The controlled platform focuses on aerodynamic and aeroelastic mechanisms under repeatable conditions, while avian takeoff also involves leg impulse, active wing morphing, sensory feedback, and species-specific predator–prey context. Within this defined scope, the robot reproduces the sequence of low- and high-angle takeoff and climb observed in some birds, providing experimental evidence for interpreting energy allocation across takeoff strategies.

The remainder of this paper is organized as follows: The “Bioinspired design of a high-thrust wing” section presents the bioinspired design of the flapping wing and its aerodynamic performance. The “Flapping–pitch effect enabling low-speed pitch control” section introduces the flapping–pitch effect and its role in low-speed pitch control. The “Whole-process simulation of different takeoff strategies” section presents full-process simulations of takeoff under different strategies and analyzes the influence of key parameters. The “Prototype and experiments” section describes the prototype implementation and flight experiments. Finally, Discussion presents the conclusions. Detailed descriptions and theoretical derivations of the FSI framework are provided in the Supplementary Materials.

## Materials and Methods

### Simulation methodology

To overcome the difficulties inherent in solving nonlinear equation systems like Navier–Stokes equations using traditional methods, this study employs the LBM [23,24], developed from molecular dynamics principles. This method is implemented within the Co-simulation-Engine multiphysics coupling platform. The coupling scheme integrates the explicit analysis step of the solid solver with the LBM fluid solver analysis step. Convergence of the coupled simulation is assessed based on the Gauss–Seidel [25] convergence criterion (Fig. 2A).

**Fig. 2.**
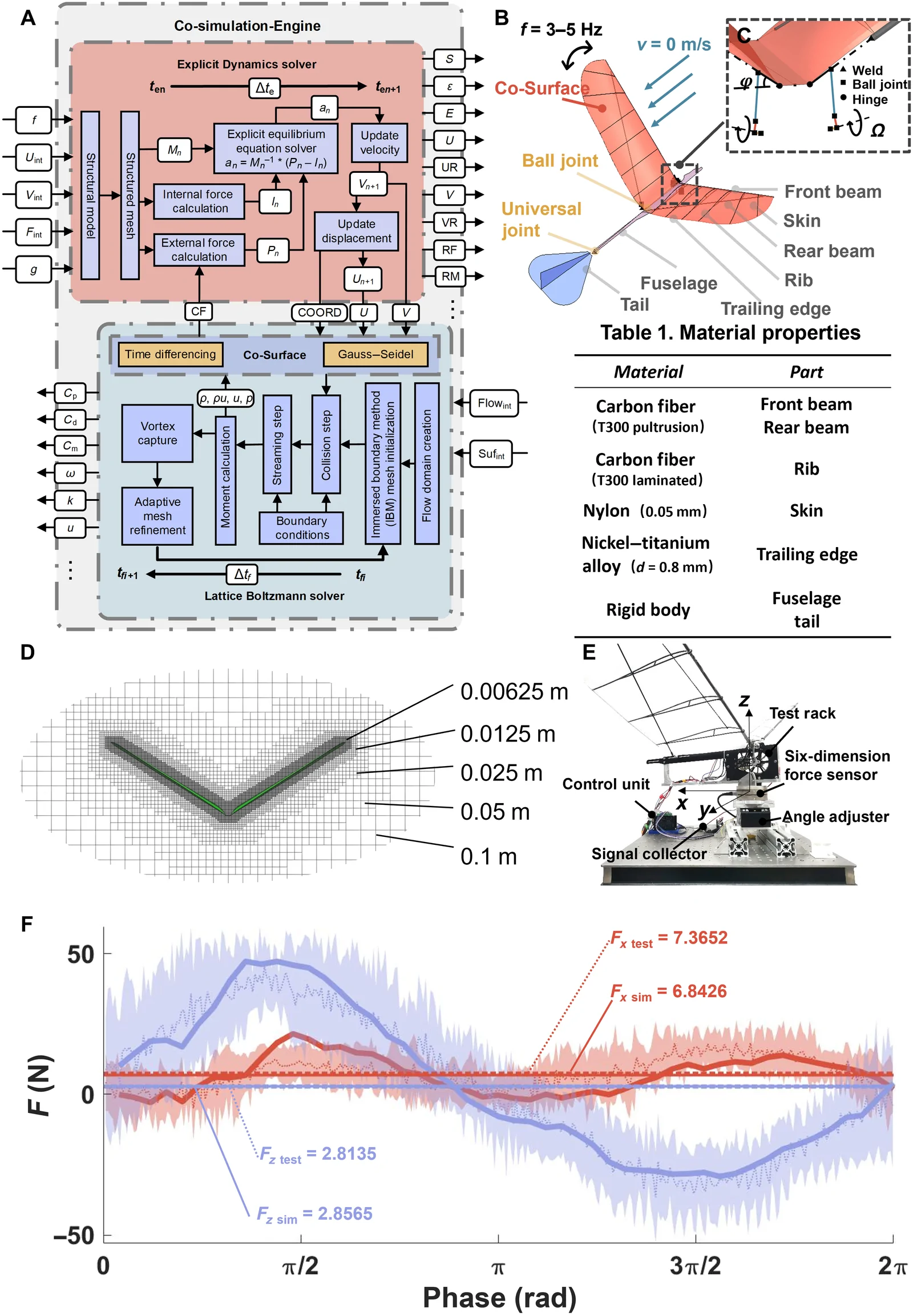
Fluid–structure interaction (FSI) simulation methodology and verification. (A) Flowchart of the FSI simulation methodology, where f, $U_{\text{int}}$, $V_{\text{int}}$, $F_{\mathrm{int}}$, and g represent the external loads acting on the structure; the initial conditions for position, velocity, and load; and the gravitational field, respectively. S, ε, E, U, UR, V, VR, RF, and RM denote the structural stress, strain, energy, displacement, angular displacement, velocity, angular velocity, reaction force, and reaction moment, respectively. $C_{p}$, $C_{d}$, and $C_{m}$ represent the pressure coefficient, drag coefficient, and moment coefficient on the coupling surface, respectively. ω, k, and u denote the vorticity, energy, and velocity of the flow field, while ${\text{Flow}}_{\mathrm{int}}$ and ${\mathrm{Suf}}_{\text{int}}$ represent the initial conditions of the flow field and the coupling surface, respectively. CF represents the nodal forces on the coupling surface, and COORD, U, and V denote the nodal coordinates, nodal displacements, and nodal velocities of the coupling surface, respectively. (B) Schematic diagram of the simulation model. (C) Simplification of the power system within the simulation model. (D) This figure illustrates the mesh resolution near the wing surface using the immersed boundary method employed in this study. A 5-level refinement scheme, with each level consisting of 6 grid layers, is applied around the wing and near the vortex cores. The refinement zones adaptively follow the motion of the wing and the vortices. (E) Six-axis force measurement platform. (F) Baseline thrust and lift data at a flapping frequency of 3 Hz under no-flow conditions, compared with simulation results. The red line represents the thrust data, and the light purple line represents the lift data. Solid lines indicate simulation results, while dashed lines represent the average values.

Physical parameters (such as displacement, velocity, and force) at the computational interface are transferred between solvers via Co-Surface elements. Within the fluid solver, the immersed boundary method [26] is employed for mesh discretization, and adaptive mesh refinement is applied to regions characterized by thick boundary layers and high vorticity gradients (Fig. 2D). This strategy effectively circumvents the challenges of mesh motion and remeshing faced by the finite-element method in large-deformation FSI computations.

### Simulation model

The simulation model uses a reasoned abstraction of the flapping-wing robot. To reduce computational cost, the wings serve as the primary flexible aeroelastic components, and the fuselage and empennage are represented as rigid bodies, concentrating structural deformation in the wing model. The complete aircraft, including the empennage, remains coupled to the same unsteady flow field, so tail loads in the full-process simulations include the wing-generated wake. Thin-walled structures such as the leading-edge spar, wing ribs, and skin are modeled using shell elements. Components are connected through no-slip bounded conditions (Fig. 2B).

The abstraction of the power-transmission mechanism is a critical aspect of model simplification. Revolute, spherical, and welded joints represent complex assemblies such as gears and cranks as equivalent rigid links. This treatment preserves the required kinematic relationships while reducing computational complexity (Fig. 2C). Material properties for all components are listed in Table 1.

The wing skin acts as the Co-Surface element. During simulation, it transfers mesh coordinates, displacements, and velocities from the solid solver to the fluid solver, and computed surface forces on the elements from the fluid solver back to the solid solver.

Intelligent adaptive mesh refinement is applied to the wake region and near-wall regions: The far-field mesh characteristic scale is 0.1 m, and the refined region mesh scale is 0.00625 m. The computational domain is a rectangular space measuring 25 m × 25 m × 5 m. The total mesh is 15 million. The characteristic scale of the skin mesh is maintained consistently with the refined near-wall fluid mesh scale to avoid numerical errors introduced by interpolation between dissimilar grids.

### Computing hardware

Computations were executed on a dual-socket AMD EPYC 9754 server (total: 256 physical cores), equipped with 768 GB of DDR5 5,600-MHz ECC RAM. For coupled FSI simulations, 64 cores were allocated to the Lattice Boltzmann fluid solver and 16 cores to the Explicit Dynamics solid solver. The computational efficiency averaged 3,480 core-hours per simulated second of physical time.

## Results and Discussion

### Bioinspired design of a high-thrust wing

The flapping-wing robot achieves sufficient aerodynamic performance to support systematic investigation of takeoff strategies.

#### Bioinspired design

To enable controlled investigation of bird-inspired takeoff strategies, we developed a meter-scale flapping-wing robot platform, Hawk 3.0 (Fig. 3A), with a wingspan of 1.82 m, a takeoff weight ranging from 955 to 1,105 g, and a maximum flapping frequency of 5 Hz. Compared with the recent research [27–30], this study features improvements in wing design and the power system, enabling enhanced flight performance.

**Fig. 3.**
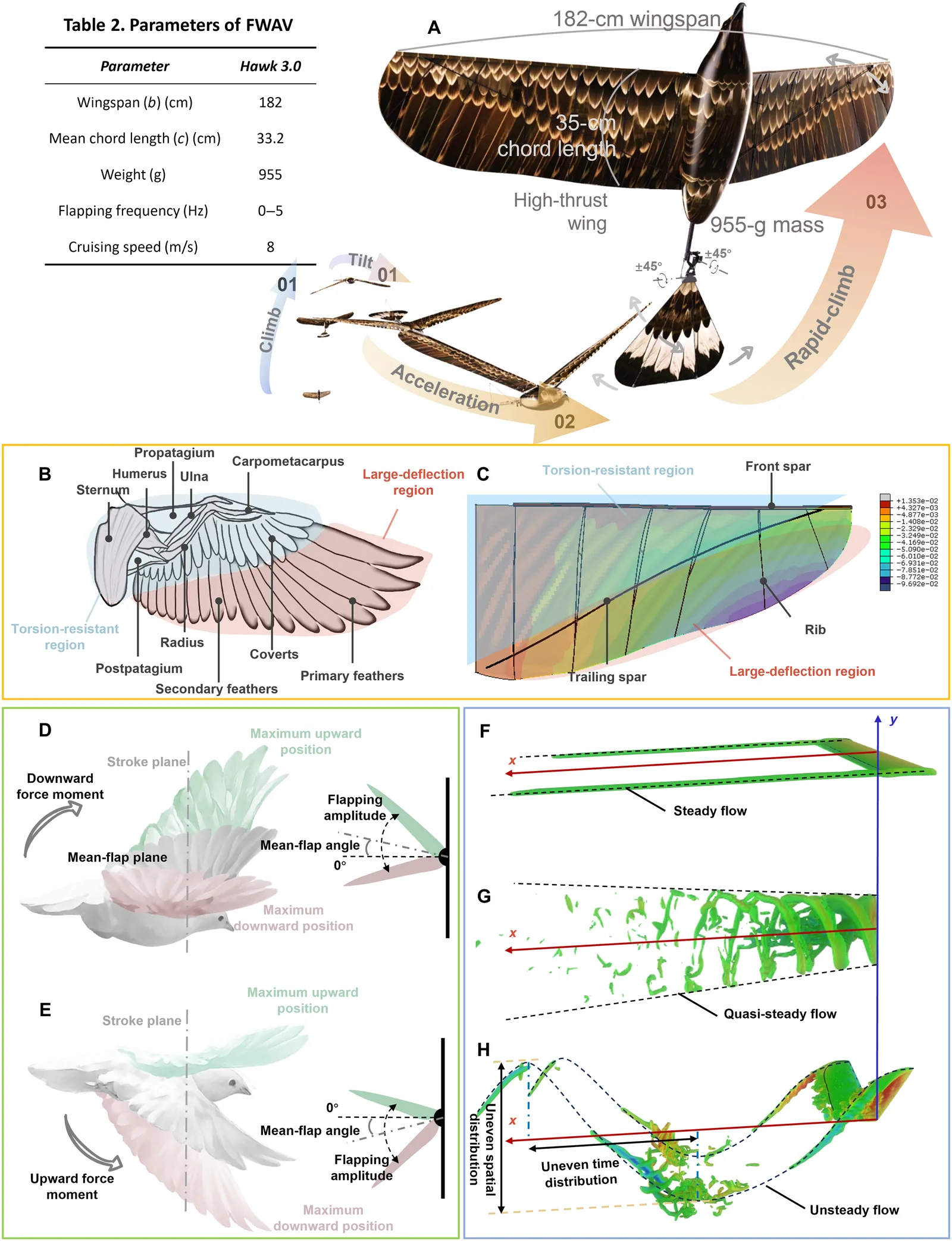
Key mechanisms enabling bird-inspired takeoff in a flapping-wing robot: high-thrust wing design and low-speed pitch control. (A) Hawk 3.0 configuration and takeoff sequence (Movie S1). (B) Structural partition of a bird wing: proximal feathered regions supported by bones and muscles exhibit high stiffness, whereas distal regions composed mainly of flight feathers are more compliant. (C) Wing deformation: the primary load-bearing region (front spar–rib–trailing spar) shows limited deformation, analogous to the stiff proximal wing; the flexible trailing region undergoes a large deformation, analogous to flight feathers (Movie S3). (D and E) Pitch control: with a constant flapping amplitude, increasing the mean-flap angle (MFA) induces a nose-down moment, while decreasing it produces a nose-up moment. (F to H) Vorticity comparison (fixed-wing/rotor/flapping wing): flapping wings generate spatiotemporally unsteady thrust, with both magnitude and line of action varying over the stroke. Thus, adjusting the MFA modulates the pitch moment by altering the thrust distribution and its moment arm.

The wing design is inspired by the stiffness distribution observed in bird wings [31,32], where relatively stiff load-bearing regions coexist with more compliant feathered regions. Following this engineering abstraction, the wing incorporates a spatial stiffness gradient: the leading-edge spar–rib framework provides the principal load path, whereas the trailing region undergoes larger passive deformation (Fig. 3B and C). This configuration supports thrust generation while maintaining structural integrity.

Finite-element stiffness comparisons confirmed that the spar-reinforced load-bearing region is consistently stiffer than the region aft of the rear spar, supporting the intended spatial stiffness gradient. The regional comparison and structural model are provided in Table S2, Fig. S4, and File S1, while the complete 18 × 18 normal-compliance and stiffness matrices are supplied in File S2.

Flight experiments demonstrate that the robot is capable of both low-angle acceleration and high-angle climb during takeoff. This capability enables controlled comparison of different takeoff strategies under identical conditions, providing a reliable experimental basis for subsequent analysis.

#### FSI simulations

To further assess flight capability, FSI simulations were conducted under no incoming flow at a flapping frequency of 5 Hz. The results reveal detailed aeroelastic deformation and flow-field evolution during flapping (Fig. 4A). At the start of the downstroke, aerodynamic loading induces torsional deformation around the leading edge and bending near the wing root. The resulting pressure difference between the lower and upper wing surfaces generates leading-edge and trailing-edge vortices, which strengthen toward the wingtip due to increasing local velocity. This indicates that the primary aerodynamic forces are concentrated near the wingtip, in contrast to fixed-wing aircraft where forces are typically distributed near the wing root.

**Fig. 4.**
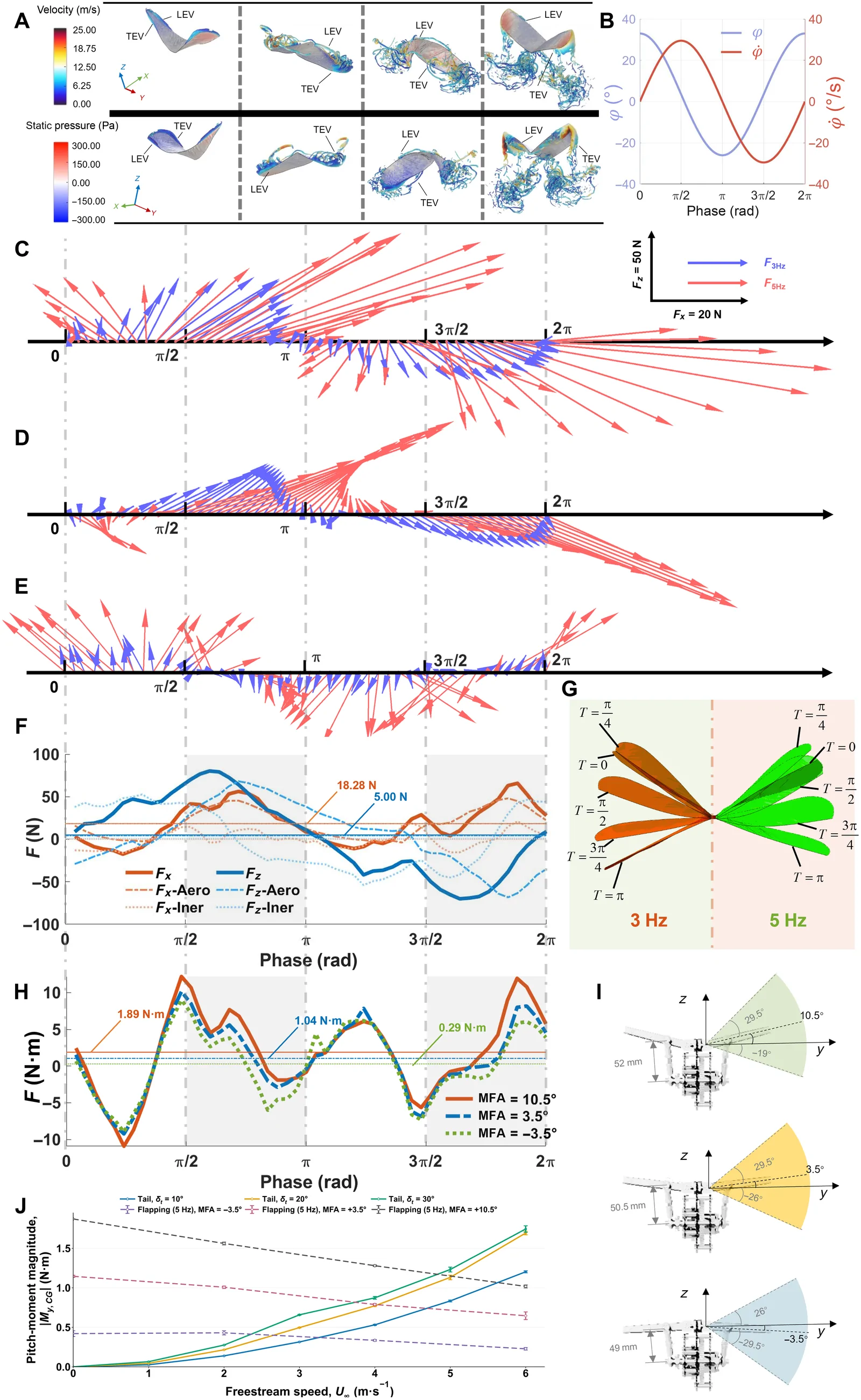
Flapping-wing aerodynamic performance analysis. (A) Flow-field visualization at a 5-Hz flapping frequency under no-flow conditions. Wing surfaces are colored by static pressure; isosurfaces in the flow field represent a vorticity magnitude of 900 s^−1^, colored by flow velocity. Row 1: Isometric view facing the wing’s upper surface. Row 2: Isometric view facing the wing’s lower surface (Movie S3). (B) Flapping angle and angular velocity of wings. (C to E) Time-domain vector representation of total forces, aerodynamic force, and inertial force at 3- and 5-Hz flapping frequencies. (F) Simulation data of lift/thrust forces at a 5-Hz flapping frequency with decomposition into flow-induced and inertial components. (G) The figure illustrates the front view of wing deformation during a downstroke at flapping frequencies of 3 Hz (left) and 5 Hz (right), with each snapshot separated by a time interval of π/4. (H) The figure displays the variation curves of pitching moment over one flapping cycle under different mean-flap angles (MFAs). A nose-up moment is defined as positive. (I) Implementation of 3 different flapping range adjustments by varying the connecting-rod length. (J) Magnitudes of pitch moment about the common aircraft center of gravity versus freestream speed. Solid curves show tail-simulation results at peak deflections of 10°, 20°, and 30°, and dashed curves show 5-Hz flapping fluid–structure interaction (FSI) results at MFA = −3.5°, +3.5°, and +10.5°. Error bars show temporal variation.

The total force generated by the flapping wing consists of aerodynamic and inertial components (Fig. 4C to E). Analysis shows that thrust is almost entirely dominated by aerodynamic forces (≈100%), while inertial forces contribute negligibly. In contrast, lift arises from both aerodynamic (≈80%) and inertial (≈20%) contributions due to asymmetric deformation between downstroke and upstroke. These results indicate that thrust generation is primarily governed by aerodynamic effects, whereas lift reflects coupled aeroelastic dynamics.

#### Experimental measurements

To assess the FSI framework, we conducted aerodynamic-force measurements and digital image correlation (DIC) deformation measurements. The zero-freestream, 3-Hz force comparison provides component-level validation relevant to the initial low-speed takeoff stage, when wing-generated forces govern release and early attitude evolution. At a flapping frequency of 3 Hz and an angle of attack of 0°, independently prescribed FSI simulations captured the periodic force characteristics, with time-averaged agreement of 92.4% for thrust and 98.5% for lift (Fig. 2F). The Supplementary Materials extend this assessment through DIC deformation and signed force-history comparisons over 4 to 12 m/s. Together with the measured flight trajectories, these component-level results provide a system-level assessment of integrated predictive accuracy.

#### Aeroelastic hysteresis in flapping-wing aerodynamics

Importantly, FSI simulations reveal a phase-dependent hysteresis between flapping kinematics and force generation. Unlike simplified theoretical models [33,34] and computational fluid dynamics predictions [35–37], where peak aerodynamic forces coincide with the maximum flapping velocity (Fig. 4B), the present results show a clear phase lag between kinematic extrema and force peaks.

FSI analysis reveals that the peaks of aerodynamic force and the zeros of inertial force do not coincide with the stroke phases of the maximum flapping velocity (π/2 and 3π/2) but instead exhibit a phase hysteresis. This hysteresis is not constant but increases gradually with higher flapping frequencies.

The hysteresis arises because the leading-edge spar is not an ideal rigid body; it bends during flapping, causing the motion phase of the wingtip to lag behind that of the wing root. As the flapping frequency increases, enhanced aerodynamic damping leads to larger deformation amplitudes of the leading-edge spar. As illustrated in Fig. 4G, which shows wing deformation at various phases during a complete downstroke at 3 and 5 Hz, it is evident that due to the bending hysteresis of the front spar, the wingtip height at T=π/4 is actually lower than that at T=0. Moreover, the wingtip lag is more pronounced at 5 Hz compared to that at 3 Hz. This occurs because the structural deformation response time is primarily determined by its natural modes and does not vary with flapping frequency, resulting in essentially the same deformation duration across different frequencies. However, since the motion period at 5 Hz is shorter than that at 3 Hz, the lag effect becomes more prominent when expressed in phase coordinates.

This aeroelastic hysteresis has 2 key implications. First, it highlights the necessity of high-fidelity FSI modeling for accurately resolving flapping-wing aerodynamics, as simplified models fail to capture phase-dependent force generation. Second, it indicates that aerodynamic forces are predominantly generated at the wingtip, where deformation-induced phase shifts are most substantial. As a result, modulation of the mean flapping angle (MFA) alters both the magnitude and effective line of action of thrust, generating different pitching moments on the fuselage.

### Flapping–pitch effect enabling low-speed pitch control

#### Low-speed pitch control challenge

Pitch control during takeoff is achieved through a flapping–pitch coupling effect that remains effective at low speeds. During the initial phase of takeoff, the airspeed is insufficient to generate effective aerodynamic forces, rendering conventional control surfaces (e.g., tails) ineffective in producing adequate pitching moments. This limitation restricts the ability to regulate climb angle and may lead to attitude instability. Therefore, achieving reliable pitch control at low speeds is essential for enabling different takeoff strategies.

In conventional flight systems, pitch control is achieved through the adjustment of the longitudinal trim condition via wing moment, tail moment, or center-of-gravity (CG) shift:

$$I_{y}\frac{dq}{dt}=M_{w}+M_{\delta }$$ (1)

where $M_{w}$ is the moment generated by the wing about the CG and $M_{\delta }$ is the moment generated by the tail.

Among these, tail-based control is typically the most efficient. However, since aerodynamic control effectiveness scales with the square of airspeed, its authority rapidly diminishes during takeoff:

$$M_{\delta }=\frac{1}{2}\rho V^{2}{\text{SC}}_{M_{\delta }}d$$ (2)

where ρ is the air density, V is the flight velocity, S is the wing reference area, $C_{M_{\delta }}$ is the aerodynamic moment coefficient of the control surface, and d is the characteristic length (e.g., mean aerodynamic chord). This implies that control effectiveness diminishes rapidly at lower airspeeds.

CG adjustment is also impractical due to mass redistribution and stability constraints:

$$C_{m_{\text{cg}}}=C_{m_{0}}+C_{m_{\alpha }}\alpha +C_{m\delta_{e}}\delta_{e}+C_{m_{T}}T=0$$ (3)

$$\frac{\partial C_{L_{\alpha }}\left(\frac{X_{\text{cg}}}{c}-\frac{X_{\text{ac}}}{c}\right)}{\partial \alpha }<0$$ (4)

The formula demonstrates the dual necessary conditions for flight trim and stability. Conversely, an aft CG position risks inducing pitch static instability, compromising flight performance during critical phases like horizontal-acceleration and rapid-climb.

#### Aerodynamic mechanism of the flapping–pitch effect

Biological flyers provide an alternative solution. Birds and insects achieve pitch control primarily through modulation of wing kinematics [38]. From an aerodynamic perspective, flapping wings generate forces whose magnitude and line of action vary dynamically over the flapping cycle, particularly at the wingtip where local velocities are highest. As a result, adjusting the MFA redistributes both the aerodynamic force and its moment arm, generating controllable pitching moments (Fig. 3D and E). This behavior differs fundamentally from that of fixed-wing and rotary-wing systems (Fig. 3F to H).

#### Flapping–pitch control authority demonstrated by FSI

Flapping–pitch effects have been demonstrated in some insect-scale and hummingbird-like flapping robots [39–41], while their quantitative use in meter-scale systems remains comparatively underexplored. Predictive analysis is particularly valuable at this scale because flexible-wing deformation, inertial loading, and unsteady aerodynamics interact strongly. We therefore evaluated the mechanism through FSI simulations of Hawk 3.0 under 3 representative stroke ranges (Fig. 4I). The preset MFA values were generated by changing the connecting-rod length, which shifted the stroke interval while preserving the overall flapping amplitude and the same basic transmission architecture. This configuration enabled controlled comparison of wing kinematics, aeroelastic loading, and the resulting pitching moment across the 3 MFA settings. The simulations establish the engineering feasibility of regulating pitch authority through linkage-defined MFA variation and provide a quantitative basis for the subsequent full-process takeoff simulations and flight-test settings. In the present prototype, each linkage setting defines a preset MFA for the corresponding flight, while future lightweight actuation can extend this mechanism to in-flight modulation.

Fig. 4H presents the variation of the pitch moment about the CG over one flapping cycle at 5 Hz for different MFAs. During the first and third quarter-cycles, where inertial forces dominate, changes in the MFA have little influence on the pitch moment. In contrast, during the second and fourth quarter-cycles, where aerodynamic forces prevail, the nose-down moment gradually strengthens as the MFA increases.

#### Airspeed-dependent transition between flapping–pitch and tail authority

We evaluated 30 simulation cases to compare tail- and flapping-induced pitch-moment authority using a common reference. All pitch moments were expressed about the aircraft CG at (141, 0, −1) mm in the flapping-coordinate frame. The dataset comprised 18 tail-simulation cases at 1 to 6 m/s and peak deflections of 10°, 20°, and 30°, together with 12 flapping FSI cases at 5 Hz; 0, 2, 4, and 6 m/s; and MFA = −3.5°, +3.5°, and +10.5°. As shown in Fig. 4J, tail moment grew strongly with dynamic pressure and followed an approximately quadratic trend before high-deflection saturation became evident. Flapping-induced pitch moment decreased as airspeed increased because aerodynamic resistance increased and net thrust diminished. Across all tested command pairs, equal-magnitude intersections occurred at approximately 2.36 to 5.63 m/s. Flapping–pitch authority dominates the near-zero and low-speed regime, and tail authority becomes progressively more influential as airspeed increases. The 5 m/s division used in the preceding tail-effect analysis lies within this transition interval and provides a representative boundary for discussing low- and high-speed tail influence.

### Whole-process simulation of different takeoff strategies

#### Whole-process simulation validation

To further validate the feasibility of the flapping–pitch effect compared to tail deflection in regulating takeoff strategies and provide references for real-world flight experiments, this study conducted comprehensive FSI simulations of the entire takeoff process under varying MFAs and different tail deflection angles. The simulation setup was as follows: The model was released via a slider with an absolute friction surface; to avoid transient effects at flapping initiation, release was set at the start of the second flapping cycle; gravity was applied in the −*z* direction; the freestream velocity was zero; the flapping frequency was 5 Hz; and the simulation duration was 3.2 s.

The pitch-angle histories in Fig. 5A and B show that MFA has a substantially larger effect on takeoff attitude than the tested tail-control patterns during the low-speed stage. Relative to the MFA = 3.5° case, MFA values of −3.5° and +10.5° produced pitch-angle changes of −60.4% and +37.6%, respectively, at the stated comparison condition.

**Fig. 5.**
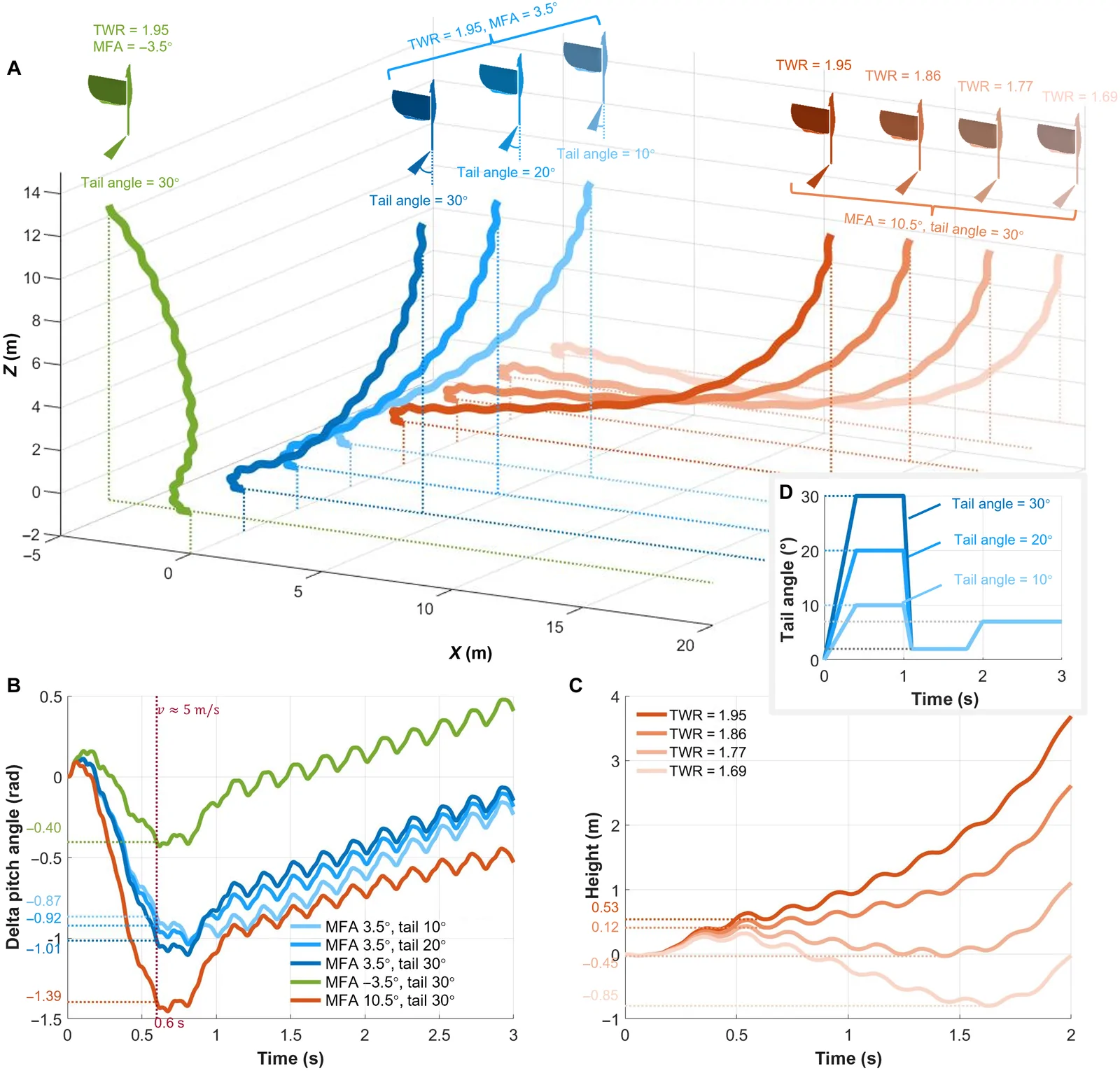
Simulation analysis of the flight process. (A) Trajectories for different mean-flap angle (MFA) values, tail-control patterns, and thrust-to-weight ratios (TWRs) (Movies S4 and S5). (B) Pitch-angle histories for different MFA values and tail-control patterns. The 3 tail patterns have peak deflections of 10°, 20°, and 30°, respectively. (C) Altitude histories for different TWRs; all curves use a peak tail deflection of 30° and MFA = 10.5° (Movie S5). (D) Prescribed tail-control patterns. Each pattern varies during takeoff and is named by its peak deflection before 1.2 s.

Tail-angle sensitivity was evaluated using 3 prescribed control patterns with peak deflections of 10°, 20°, and 30° (Fig. 5A, B, and D). At the stated 5 m/s comparison condition, the pitch angles for the 20° and 10° patterns differed by 8.9% and 13.9%, respectively, from the 30° pattern. The 30° pattern provided the largest restoring moment, while Fig. 4J shows that the incremental gain diminished as tail deflection increased, indicating a high-deflection aerodynamic saturation trend. We therefore selected 30° to balance pitch-recovery authority, the observed saturation trend, and flight-test margin. The larger recovery margin also reduced flight-test risk for the larger-MFA conditions identified by the full-process simulations.

In summary, for this study, we adopted a combined approach utilizing both the flapping–pitch effect and tail control to adjust the takeoff strategy in the flight experiments. Specifically, adjusting the length of the linkage in the power system changes the MFA. During the initial phase of takeoff, the tail does not participate in control; the takeoff attitude is passively altered solely by the nose-down moment generated by the wings. As flight speed increases, the tail’s control authority gradually becomes capable of balancing the nose-down tendency induced by the flapping–pitch effect, thereby enabling the robot to maintain its climb angle. In the full-process takeoff simulations, the wings, fuselage, and tail are resolved within the same unsteady flow field and coupled body-motion model; the resulting tail loads therefore include the influence of the flapping-wing wake.

#### Decisive role of TWR

The TWR is another critical parameter for achieving takeoff. Although takeoff in this study was initiated from an elevated auxiliary support structure for safety considerations, we strictly maintained that the flight altitude must not fall below the initial height during the entire takeoff process. This is because, in real natural environments, such a drop could imply that the bird has already descended back to the ground.

To determine the minimum TWR required for takeoff and establish quantitative design benchmarks, FSI simulations were conducted across varying TWR conditions. By adjusting virtual counterweights within the simulation model—modifying mass magnitude and position while maintaining constant CG—precise TWR modulation was achieved. Simulations spanned 4 takeoff masses: 955, 1,005, 1,055, and 1,105 g, corresponding to TWRs of 1.95, 1.86, 1.77, and 1.69, respectively. Since a larger average MFA corresponds to a lower takeoff angle, this analysis was conducted with an MFA of 10.5°, as shown in Fig. 5A and C.

The variation in TWR substantially affects the maximum flight altitude during the climb phase and the subsequent kinetic energy accumulation after transitioning to level flight. As the TWR gradually decreases, the flapping-wing robot’s climb becomes more sluggish, leading to a progressive decline in the maximum flight altitude attained during the climb phase. Concurrently, due to insufficient flight speed, the robot experiences inadequate lift after transitioning to level flight and cannot maintain stable flight (Fig. 5C). This results in the vehicle continuously diving until sufficient gravitational potential energy is converted into kinetic energy to sustain level flight. When the TWR drops below 1.77, although the flapping-wing robot can still achieve a minor initial climb during takeoff, it inevitably loses altitude below the initial takeoff altitude during the level acceleration phase.

### Prototype and experiments

#### Development of the self-takeoff flight prototype Hawk 3.0

Hawk 3.0 is a flapping-wing robot resulting from our design effort to balance flight performance against weight reduction (Fig. 6A). It comprises 4 core subsystems: a high-thrust wing, a 2-degrees-of-freedom empennage, a power system, and an avionics system. To meet the TWR design requirements, we implemented lightweight design across all components and prudently optimized structural parameters to avoid weight increases associated with overdesign. Through further efforts to reduce battery weight (utilizing a high-voltage lithium battery weighing only 52 g), the final takeoff weight was controlled at 955 g. This resulted in a prototype TWR of 1.95, surpassing the design target of TWR.

**Fig. 6.**
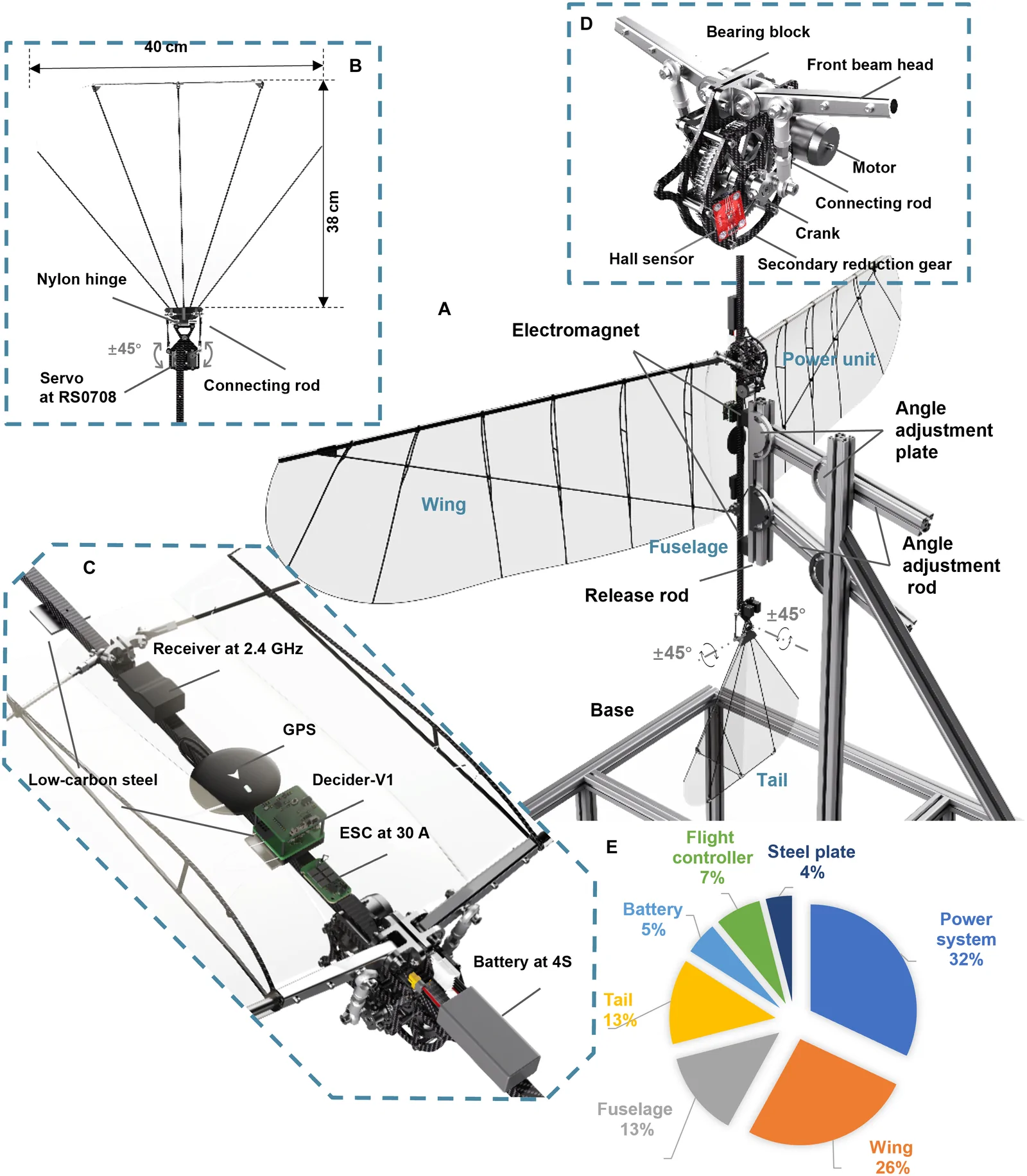
Hawk 3.0 prototype development. (A) The image depicts Hawk 3.0 positioned on the auxiliary support structure prior to takeoff. (B) Tail actuation mechanism. A parallel mechanism driven by dual servo motors enables torque superposition, reducing requirements for servo size and weight. (C) Avionics system. Includes the battery, electronic speed control (ESC), flight controller, GPS, and receiver. Low-carbon steel plates interface with electromagnets. (D) Power system. Comprises a motor, reduction gears, and a linkage mechanism. Hall sensors simultaneously monitor flapping frequency and phase. (E) Weight distribution breakdown of prototype components.

The airframe extensively uses T300 carbon-fiber composite materials. Wing ribs are computer-numerical-control-machined from 2.5-mm carbon-fiber sheets, whereas spars and rods primarily use pultruded carbon-fiber profiles. The wing skin is bonded to the skeletal structure using 5800 instant-curing adhesive and 3M high-strength double-sided tape. Flight power is supplied by a 500-mAh, 4S high-voltage LiPo battery with a maximum voltage of 17.4 V and a discharge rating of 100 C. The power system uses a 2216-type, 1,400-kV brushless dc motor.

The empennage employs a fully movable design (Fig. 6B). The horizontal tail features an isosceles triangular configuration mimicking avian tails. To provide basic yaw stability and control authority, an integrated right-triangular vertical stabilizer (area: 280 cm^2^/2.8 dm^2^) is mounted atop the horizontal tailplane. Two parallel RS0708 servos drive the tail assembly, enabling ±45° deflection around their horizontal and vertical hinges for pitch and yaw control, respectively. The internal structure consists of a spatial truss made of 2-mm carbon-fiber rods for enhanced rigidity, covered with the same P14 parachute fabric as the wings.

The power system not only serves as the core driver for wing flapping but also plays a crucial role in modulating takeoff trajectories through wing–body coupling effects. Motor output is transmitted via a 2-stage gear reducer (reduction ratio: 55.7:1) to the crank. This power module has a maximum input power of 500 W and achieves a maximum flapping frequency of 5 Hz. The module is rigidly bolted to a 10-mm carbon-fiber square tube fuselage. The flapping motion is generated by a spatial 4-bar linkage mechanism comprising high-precision spherical hinges and the crank (Fig. 6D). To prevent issues like sudden increases in transmission ratio, loss of mechanical precision, and exacerbated dead-center effects at large swing angles, the design limits the maximum flapping angle to 59°. Adjusting the linkage length allows modification of the MFA while maintaining a constant flapping range: −33° to 26° (MFA: −3.5°), −26° to 33° (MFA: 3.5°), and −19° to 40° (MFA: 10.5°) (Fig. 4I).

We utilize a self-developed flight controller, Decider-V1, integrated with a 6-axis (gyroscope and accelerometer) microelectromechanical system motion-tracking device (MPU6050) on the flight platform (Fig. 6C). This sensor provides 6-axis MotionFusion data at a 500-Hz digital output rate, used to obtain the drone’s motion states. A high-precision NEO3PRO GPS measures the drone’s position. The power component monitors voltage and current to evaluate power consumption. An A3144 Hall effect sensor, rigidly mounted on the power system, captures wing position and rotational speed (Fig. 6D). The weight distribution breakdown of its components is shown in Fig. 6E.

Before takeoff, the flapping-wing aircraft needs to flap at high power in a stable initial pitch-angle state, which generates a maximum aerodynamic load of more than 90 N. At the same time, the release device should not add too much extra structure to the prototype. To support the prototype before takeoff, the auxiliary launch platform uses a 30-mm aluminum-profile truss base (1.5 m × 1 m × 0.5 m), an angle-adjustable support frame, and electromagnetic release rods. The release angle is configurable from 0° to 120°. Low-carbon steel plates on the fuselage are attached to 2 electromagnets, and the robot is released by de-energizing the magnets when the flapping frequency reaches 5 Hz and the wings reach the top-dead-center position.

#### Flight experiments

Under the tested MFA, tail-control, TWR, and open-loop settings, low-angle takeoff increases flight distance while reaching a comparable altitude at the fixed 3-s evaluation time.

Flight experiments were conducted outdoors under nearly wind-free conditions (Fig. 7A), with 3 tests completed for each preset MFA (−3.5°, 3.5°, and 10.5°). All tests used the same launch platform, battery-voltage range, CG position, initial attitude, release phase, open-loop tail command, and field environment. Each test began at a pitch angle of 90°, and release occurred when the flapping frequency reached 5 Hz and the wings reached the top-dead-center position; the auxiliary platform contributed zero propulsive energy. The Decider-V1 controller applied the tail pattern used in simulation, with a peak deflection of 30°. This pattern provided a strong pitch-recovery margin and operated near the onset of high-deflection aerodynamic saturation, supporting robust execution of the larger-MFA tests. The common 3-s endpoint was applied to all trials. By this time, the release transient had ended, the prototype had established sufficient speed and altitude for subsequent maneuvers, and altitude-, speed-, and energy-related states had entered a comparable range while retaining the accumulated distance history of each strategy.

**Fig. 7.**
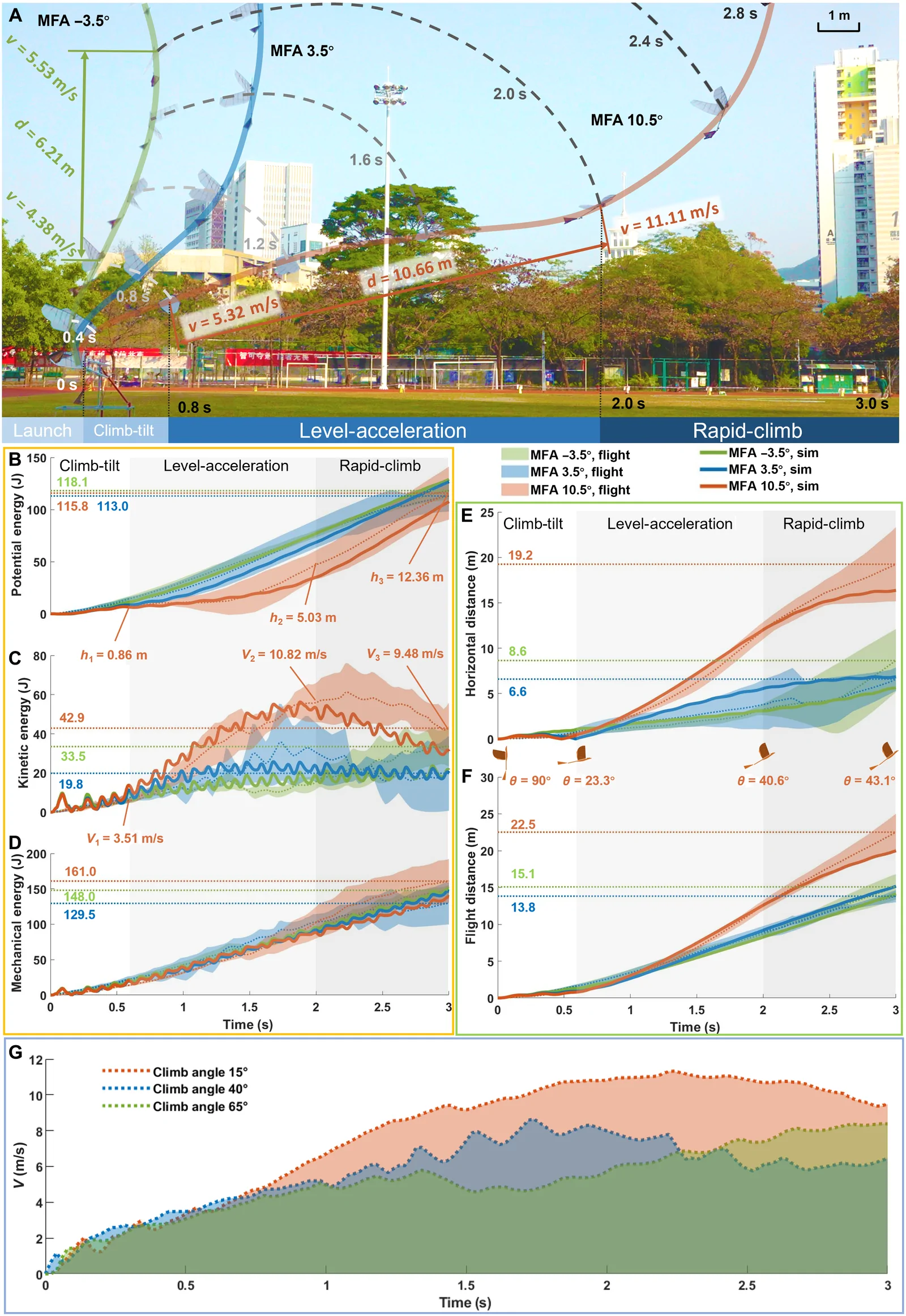
Flight experiments (Movie S6). (A) Silhouette sequences of self-takeoff for the 3 mean-flap angle (MFA) settings. (B to D) Energy-allocation histories during takeoff. Three trials were conducted for each MFA; dashed curves show the mean trajectories, and shaded regions show the trial-to-trial envelope. The horizontal dashed line marks the value at the fixed 3-s engineering evaluation time. (E and F) Mean horizontal and total flight distances, respectively, evaluated at 3 s; the aircraft silhouettes indicate the 15° low-angle condition. (G) Speed histories for the 3 takeoff strategies. The area under each speed curve gives the corresponding traveled distance.

The flight tests reproduced the principal attitude, energy-allocation, speed, and trajectory trends obtained from the simulations (Fig. 7A to C). Agreement across these coupled quantities provides system-level validation of the simulation framework and confirms its ability to capture the principal takeoff dynamics and strategy-dependent trends. As MFA increased from −3.5° to +10.5°, the measured takeoff climb angle decreased from approximately 65° to 15°, with the intermediate MFA = 3.5° condition producing approximately 40°.

As shown in Fig. 7B, the larger-angle strategy had a higher climb rate during approximately the first 2 s and accumulated gravitational potential energy faster. Fig. 7C shows that the low-angle strategy accelerated more rapidly. Under similar input-power conditions, these paths represent different temporal allocations between kinetic and potential energy. By 3 s, their altitude, speed, and mechanical energy states had converged to a comparable range, providing a common posttransient state for evaluating the distance accumulated along each path.

At the fixed 3-s evaluation time, traveled distance provides an engineering measure of separation from the release point. We compared horizontal distance (Fig. 7E) and total distance (Fig. 7F) using mean trajectories from 3 trials per MFA condition. Relative to the mean 65° high-angle strategy, the mean 15° low-angle strategy increased total distance by 49% and horizontal distance by 123%. The high-angle strategy established an early climb advantage, and the low-angle strategy accumulated greater speed and distance before converting part of its kinetic energy into altitude.

The advantage of the low-angle takeoff strategy in flight distance stems from its temporal allocation strategy for kinetic and potential energy. As shown in Fig. 7F, when adopting a low-angle takeoff strategy, the robot rapidly reduces its pitch angle to 23.3° and transitions into a horizontal-acceleration phase. At this point, thrust is primarily used to overcome air drag, which is much smaller than gravity, allowing the robot to achieve greater acceleration. In contrast, the thrust in a high-angle climbing strategy continuously counteracts gravity, resulting in smaller acceleration. Consequently, the low-angle strategy maintains a higher flight speed throughout the entire takeoff process.

As illustrated in Fig. 7A, the low-angle takeoff process can be divided into 3 stages based on attitude characteristics: climb-tilt, horizontal-acceleration, and rapid-climb. At 0.8 s after takeoff, the flight speed of the low-angle strategy reached 5.32 m/s, similar to the 4.38 m/s of the high-angle strategy; by 2 s, its speed had reached 11.11 m/s, approximately twice that of the high-angle strategy. After 2 s, although the tail deflection setting caused the robot to begin climbing and decelerating, the accumulated speed advantage still allowed it to complete more altitude gain in a shorter time.

Fig. 7G shows the variation of flight speed over time under different takeoff strategies. The area enclosed by the speed curve and the horizontal axis corresponds to the flight distance, which helps in understanding the strategic differences from a calculus perspective: The difference in the area enclosed by the velocity curves directly reflects the relative advantage in flight distance. This quantitative relationship further confirms the previous analysis of the low-angle takeoff strategy: by allocating more energy to the horizontal-acceleration phase and maintaining a higher speed over a longer period, the low-angle takeoff strategy effectively expands this integral area, thereby achieving a greater flight distance within the same time frame. This also explains, from a kinematic perspective, why substantialdifferences in flight distance arise from different temporal energy-allocation strategies, even when the mechanical energy levels are similar. Of course, the flight speed is limited and depends on the bird’s flight capability. Therefore, the maximum speed during takeoff may also serve as a metric for comparing the performance of birds or aircraft. Notably, when taking off from a higher position, birds sometimes adopt a negative takeoff angle, leveraging gravity to accelerate and achieve greater flight distance.

## Discussion

This study provides a system-level investigation of takeoff strategies by integrating controlled robotic experiments with high-fidelity aeroelastic simulations. The results show that different takeoff strategies fundamentally correspond to distinct temporal allocations of kinetic and potential energy, which directly govern acceleration, climb, and flight distance.

A key finding is that under the tested platform and control settings, the low-angle strategy gains more distance while reaching a comparable flight state at 3 s. The low-angle path prioritizes early acceleration and later converts part of the accumulated kinetic energy into altitude. The high-angle path provides a higher early climb rate. This engineering trade-off supplies a physical basis for interpreting how some birds may distribute energy between acceleration and climb during escape takeoff.

Importantly, this study identifies and quantifies a flapping–pitch coupling effect that provides pitch authority in the evaluated near-zero and low-speed takeoff regime. Successful execution of the simulated takeoff strategies depends strongly on TWR, so the present prototype prioritizes a lightweight preset MFA linkage. Consequently, MFA remains fixed during each flight. Future lightweight servo-driven linkages or adjustable wing-mount mechanisms could add in-flight MFA control, enabling richer trajectories and birdlike agility. For missions with a wider mass margin, MFA modulation can serve as a complementary pitch-control channel alongside the tail. Tail simulations provide the speed-deflection authority map, and the full-process simulations include the unsteady wing wake acting on the tail. Together, the results show that flapping–pitch authority is strongest near takeoff, with the tail becoming progressively more influential as airspeed increases.

The coupled analysis links measured wing deformation and aerodynamic loading to body motion and trajectory evolution. Agreement at the component and flight levels shows that the framework captures how aeroelastic forces accumulate into vehicle-level performance. This integrated capability supports design and control evaluation for meter-scale flapping-wing takeoff.

The FSI results further reveal pronounced aeroelastic hysteresis in flapping-wing aerodynamics, where structural deformation introduces a phase lag between kinematics and force generation. This phase relationship changes force magnitude and timing, highlighting the value of explicit structural coupling. The result supports high-fidelity aeroelastic modeling for flapping-wing flight dynamics and future design and control optimization.

Species-specific variations in the observed low-angle preference can be interpreted through relative performance. Birds with high power-to-weight ratios, such as swifts [42] and skylarks [43], may possess climb capabilities comparable to or greater than those of their predators, making steep ascent effective in some contexts. Takeoff strategy therefore emerges from the interaction of morphology, power-to-weight ratio, habitat, and predator–prey performance. The low-angle distance advantage identified by the robotic platform provides a controlled physical mechanism for interpreting escape behavior.

Leg-assisted takeoff adds an initial impulse that can alter early acceleration and trajectory, and biological studies apply different definitions of takeoff completion [15,44–47]. Biological takeoff combines this leg impulse with active wing morphing, sensory feedback, and species-specific predator–prey context. The controlled platform follows the continuous transition to sustained flight and focuses on aerodynamic and aeroelastic mechanisms under repeatable conditions. Within this defined scope, the robot reproduces the behavioral sequence of low- and high-angle takeoff and climb observed in some birds, providing experimental evidence for understanding energy allocation and strategy during takeoff and climb.

In summary, this work combines a meter-scale robotic platform with an LBM-based 2-way FSI framework to compare flapping-wing takeoff strategies under controlled conditions. The results identify flapping–pitch coupling as an effective source of low-speed pitch authority and show how takeoff angle changes the temporal allocation of kinetic and potential energy. By the 3-s evaluation point, all strategies had established flight states suitable for subsequent maneuvers and reached comparable altitude-, speed-, and energy-related quantities; the low-angle strategy had accumulated 49% more total distance through earlier kinetic energy allocation. These findings support bioinspired aircraft design and provide a physical framework for interpreting selected avian takeoff observations.

## Data Availability

All data are available in the main text or the Supplementary Materials.

## Supplementary Materials

Supplementary 1 Supplementary Materials Figs. S1 to S4 Tables S1 and S2 Files S1 and S2 Movies S1 to S6 Data S1 to S3 References [48–51]

## References

[1] Kullberg C, Lafrenz M. Escape take-off strategies in birds: The significance of protective cover. Behav Ecol Sociobiol. 2007;61(10):1555–1560.

[2] Hedenstrom A, Rosén M. Predator versus prey: On aerial hunting and escape strategies in birds. Behav Ecol. 2001;12(2):150–156.

[3] Lind J, Fransson T, Jakobsson S, Kullberg C. Reduced take-off ability in robins (*Erithacus rubecula*) due to migratory fuel load. Behav Ecol Sociobiol. 1999;46(1):65–70.

[4] Burns J, Ydenberg R. The effects of wing loading and gender on the escape flights of least sandpipers (*Calidris minutilla*) and western sandpipers (*Calidris mauri*). Behav Ecol Sociobiol. 2002;52(2):128–136.

[5] Kullberg C, Fransson T, Jakobsson S. Impaired predator evasion in fat blackcaps (*Sylvia atricapilla*). Proc Biol Sci. 1996;263(1377):1619–1756.

[6] Lind J, Kaby U, Jakobsson S. Split-second escape decisions in blue tits (*Parus caeruleus*). Naturwissenschaften. 2002;89(9):420–423. 10.1007/s00114-002-0345-8 12435096

[7] Shen Y, Xu Y, Huang W, Shang C, Shi Q. Effect of coupled wing motion on the aerodynamic performance during different flight stages of pigeon. Cyborg Bionic Syst. 2025;6:200. 10.34133/cbsystems.0200 PMC12087764 40391169

[8] Berg AM, Biewener AA. Kinematics and power requirements of ascending and descending flight in the pigeon (*Columba livia*). J Exp Biol. 2008;211(7):1120–1130. 10.1242/jeb.010413 18344487

[9] Norberg RÅ, Norberg UM. Take-off, landing, and flight speed during fishing flights of *Gavia stellata* (Pont.). Ornis Scand. 1971;2(1):55–67.

[10] Kullberg C, Jakobsson S, Fransson T. Predator-induced take-off strategy in great tits (*Parus major*). Proc Biol Sci. 1998;265(1406):1659–1664.

[11] Ma Z, Zhao J, Yu L, Liang L, Liu Z, Gu Y, Wu J, Wang W, Yan S. Piezoelectric energy harvesting from the thorax vibration of freely flying bees. Cyborg Bionic Syst. 2025;6:210. 10.34133/cbsystems.0210 PMC11861424 40017697

[12] Phan HV, Floreano D. A twist of the tail in turning maneuvers of bird-inspired drones. Sci Robot. 2024;9(96):eado3890. 10.1126/scirobotics.ado3890 39565865

[13] Tu Z, Fei F, Zhang J, Deng X. An at-scale tailless flapping-wing hummingbird robot. I. Design, optimization, and experimental validation. IEEE Trans Robot. 2020;36(5):1511–1525.

[14] Chang E, Chin DD, Lentink D. Bird-inspired reflexive morphing enables rudderless flight. Sci Robot. 2024;9(96):eado4535. 10.1126/scirobotics.ado4535 39565868

[15] Li X, Zhang Z, Dunkin F, Li H, Xu J, Liu J. Large-sized ornithopters achieve continuous flight through jump-assisted takeoff. NPJ Robot. 2026;4(1):4.

[16] Chen A, Song B, Liu K, Wang Z, Xue D, Qi H. Flapping-wing robot achieves bird-style self-takeoff by adopting reconfigurable mechanisms. Sci Adv. 2025;11(36):eadx0465. 10.1126/sciadv.adx0465 PMC12407090 40901965

[17] Chen A, Song B, Wang Z, Xue D, Liu K. A novel actuation strategy for an agile bio-inspired FWAV performing a morphing-coupled wingbeat pattern. IEEE Trans Robot. 2021;39(1):452–469.

[18] Chen MW, Wu JH, Sun M. Generation of the pitch moment during the controlled flight after takeoff of fruitflies. PLOS ONE. 2017;12(3): Article e0173481. 10.1371/journal.pone.0173481 PMC5351871 28296907

[19] Berg Robertson AM, Biewener AA. Muscle function during takeoff and landing flight in the pigeon (*Columba livia*). J Exp Biol. 2012;215(23):4104–4141. 10.1242/jeb.075275 22972885

[20] Vo-Doan TT, Dung VT, Sato H. A cyborg insect reveals a function of a muscle in free flight. Cyborg Bionic Syst. 2022;2022: Article 9780504. 10.34133/2022/9780504 PMC9494732 36285304

[21] Hudson OA, Fanni M, Ahmed SM, Sameh A. Autonomous flight take-off in flapping wing aerial vehicles. J Intell Robot Syst. 2020;98(1):135–152.

[22] Hashimoto N, Suzuki R, Kikuchi K. CFD analysis of takeoff from a water surface for an insect-scale aerial/aquatic robot. J Robot Mechatron. 2024;36(5):1122–1133.

[23] Geier M, Greiner A, Korvink JG. A factorized central moment lattice Boltzmann method. Eur Phys J Spec Top. 2009;171(1):55–61.

[24] Premnath KN, Banerjee S. On the three-dimensional central moment lattice Boltzmann method. J Stat Phys. 2011;143(4):747–794.

[25] Bai ZZ, Wang L, Wu WT. On convergence rate of the randomized Gauss-Seidel method. Linear Algebra Appl. 2021;611:237–252.

[26] D’Humières D. Multiple-relaxation-time lattice Boltzmann models in three dimensions. Philos Trans A Math Phys Eng Sci. 2002;360(1792):437–451. 10.1098/rsta.2001.0955 16214687

[27] Pan E, Xu H, Yuan H, Peng J, Xu W. HIT-Hawk and HIT-Phoenix: Two kinds of flapping-wing flying robotic birds with wingspans beyond 2 meters. Biomim Intell Robot. 2021;1: Article 100002.

[28] Wang H, Xu W, Hou L, Pan E. Ospreys-inspired self-takeoff strategy of an eagle-scale flapping-wing robot: System design and flight experiments. In: *2024 IEEE international conference on robotics and automation (ICRA)*. New York (NY): IEEE; 2024. p. 3669–3675.

[29] Wang H, Gong Z, Pan E, Xu W. Eagle-scale flapping-wing robot with aggressive roll maneuverability: Bio-inspired actuation, fluid-structure interaction simulation and flight experiment. In: *2025 IEEE/RSJ international conference on intelligent robots and systems (IROS)*. New York (NY): IEEE; 2025. p. 15734–15739.

[30] Zhang S, Shen Y, Huang W, Shang C, Chen W, Shi Q. Modeling and design of a pigeon-inspired robot with passively bending wings. IEEE Robot Autom Lett. 2023;8(8):5251–5258.

[31] Crandell KE, Tobalske BW. Aerodynamics of tip-reversal upstroke in a revolving pigeon wing. J Exp Biol. 2011;214(11):1867–1873. 10.1242/jeb.051342 21562173

[32] Usherwood JR. The aerodynamic forces and pressure distribution of a revolving pigeon wing. Exp Fluids. 2009;46(5):991–1003. 10.1007/s00348-008-0596-z PMC3380271 22736891

[33] Platzer MF, Jones KD, Young J, Lai JCS. Flapping wing aerodynamics: Progress and challenges. AIAA J. 2008;46(9):2136–2149.

[34] Taha HE, Hajj MR, Nayfeh AH. Flight dynamics and control of flapping-wing MAVs: A review. Nonlinear Dyn. 2012;70(2):907–939.

[35] Bode-Oke AT, Zeyghami S, Dong H. Aerodynamics and flow features of a damselfly in takeoff flight. Bioinspir Biomim. 2017;12(5):56006. 10.1088/1748-3190/aa7f52 28699620

[36] Wu B, Huang C, Li X, Xu J, Liu S, Lam J, Wang Z, Dai J. Rhythm-based power allocation strategy of bionic tail-flapping for propulsion enhancement. IEEE Trans Robot. 2025;41:3986–4004.

[37] Yin Z, Zhou C, Liao X, Wang X, Zhang Z, Cheng L, Fan J, Wang J. SSDVM: A sliding strip discrete vortex method applied to hydrodynamic calculations for robotic fish. IEEE Trans Robot. 2025;41:6384–6402.

[38] Fang X, Wen Y, Gao Z, Gao K, Luo Q, Peng H, Du R. Review of the flight control method of a bird-like flapping-wing air vehicle. Micromachines. 2023;14(8):1547. 10.3390/mi14081547 PMC10456679 37630083

[39] Bena RM, Yang X, Calderón AA, Pérez-Arancibia NO. High-performance six-DOF flight control of the Bee^++^: An inclined-stroke-plane approach. IEEE Trans Robot. 2023;39(2):1668–1684.

[40] Chukewad YM, James J, Singh A, Fuller S. RoboFly: An insect-sized robot with simplified fabrication that is capable of flight, ground, and water surface locomotion. IEEE Trans Robot. 2021;37(6):2025–2040.

[41] Tu Z, Fei F, Deng X. Bio-inspired rapid escape and tight body flip on an at-scale flapping wing hummingbird robot via reinforcement learning. IEEE Trans Robot. 2021;37(5):1742–1751.

[42] Hedenström A, Rosén M, Akesson S, Spina F. Flight performance during hunting excursions in Eleonora’s falcon *Falco eleonorae*. J Exp Biol. 1999;202(Pt 15):2029–2039. 10.1242/jeb.202.15.2029 10393818

[43] Cresswell W. Song as a pursuit-deterrent signal, and its occurrence relative to other anti-predation behaviours of skylark (*Alauda arvensis*) on attack by merlins (*Falco columbarius*). Behav Ecol Sociobiol. 1994;34(3):217–223.

[44] Earls KD. Kinematics and mechanics of ground take-off in the starling *Sturnis vulgaris* and the quail *Coturnix coturnix*. J Exp Biol. 2000;203(4):725–739. 10.1242/jeb.203.4.725 10648214

[45] Tobalske BW, Altshuler DL, Powers DR. Take-off mechanics in hummingbirds (Trochilidae). J Exp Biol. 2004;207(8):1345–1352. 10.1242/jeb.00889 15010485

[46] Xu Y, Zhang W, Peng L, Zhou Q, Li Q, Shi Q. A locust-inspired robot capable of continuous crawl–jump–gliding locomotion with optimized transitional control. IEEE Trans Robot. 2025;41:220–235.

[47] Xu Y, Jin Y, Zhang W, Si Y, Zhang Y, Li C, Shi Q. Design and optimization of a miniature locust-inspired stable jumping robot. IEEE Robot Autom Lett. 2023;8(8):4673–4680.

[48] Chen S, Li H, Guo S, Tong M, Ji B. Unsteady aerodynamic model of flexible flapping wing. Aerosp Sci Technol. 2018;80:354–367.

[49] Takizawa K, Tezduyar TE, Kostov N. Sequentially-coupled space–time FSI analysis of bio-inspired flapping-wing aerodynamics of an MAV. Comput Mech. 2014;54(2):213–233.

[50] Mendelson L, Techet AH. Quantitative wake analysis of a freely swimming fish using 3D synthetic aperture PIV. Exp Fluids. 2015;56(7):135.

[51] Ducros F, Nicoud F, Poinsot T. Wall-adapting local eddy-viscosity models for simulations in complex geometries. Int J Numer Methods Fluids. 1998;6:293–299.

