# Supplementary material for: Birdlike Takeoff Strategies Enabled by Flapping–Pitch Effect in a Meter-Scale Flapping-Wing Robot
Source: Cyborg Bionic Syst. 2026 Oct 16;7:0714. doi: 10.34133/cbsystems.0714 (PMC13672962; doi:10.34133/cbsystems.0714)
Supplement: Supplementary file 1 [file cbsystems.0714.f1.zip › SUPPLEMENTARY MATERIALS.docx]

SUPPLEMENTARY MATERIALS

- Supplementary Materials.
- Fig. S1. Comparison between wind tunnel DIC measurements and FSI predictions.
- Fig. S2. Comparison of aerodynamic forces between wind tunnel measurements and FSI predictions.
- Fig. S3. Flight Control PCB Design
- Fig. S4. Wing structural model and regional stiffness distribution.
- Supplementary File S1. Assembled-wing three-dimensional model in STEP (.stp) format.
- Supplementary File S2 Wing stiffness matrices.
- Movie S1. Low-angle Takeoff of the Flapping-Wing Robot, Illustrating Rapid Acceleration and Subsequent Climb.
- Movie S2. Representative Takeoff Behaviors of Different Bird Species During Escape from Predators.
- Movie S3. Fluid-Structure Interaction (FSI) Simulation of Wings.
- Movie S4. Takeoff Simulations with Variable Tail Angles and Mean-Flap Angle.
- Movie S5. Takeoff Simulations with Different Thrust-to-Weight Ratios(TWRs).
- Movie S6. Takeoff Flight Experiments with Variable Mean-Flap Angle.
- Data S1. Wing Test and Simulation
- Data S2. Comparison between wind tunnel DIC measurements and FSI predictions.
- Data S3. Comparison of aerodynamic forces between wind tunnel measurements and FSI predictions.

**Supplementary Materials**

***A. Multi-physics coupled simulation method and grid independence verification***

Although simulation researchers have made numerous attempts at FSI analysis for flapping wings, existing methods either require pre-acquisition of structural deformation data or rely on simplified models for fluid simulations. Current approaches exhibit limitations in adequately accounting for wing deformation under coupled FSI effects and in resolving the influence of structural stiffness on aerodynamic performance. The application of FSI simulation to flapping-wing design currently faces the following key challenges:

Thick Boundary Layers and Complex Vortex Structures: The boundary layer thickness on flapping wings is often on the same order of magnitude as the airfoil chord length

[33]. The flow field exhibits complex vortex distributions, frequently involving the alternating shedding of large-scale leading-edge and trailing-edge vortices. This renders traditional FSI methods based on the Finite Element Method (FEM) and incompressible Navier-Stokes (N-S) equations largely ineffective for studying flapping-wing flows and engineering design. These methods are hampered by: Difficulties in mesh generation, High complexity in dynamic mesh tracking and refinement, Low efficiency in solving nonlinear equations, and Sensitivity of convergence to model parameters.

Strongly Nonlinear FSI Effects: Flapping-wing aerial vehicles primarily operate at low Reynolds numbers. Their lift and thrust generation fundamentally arises from the plunging motion and twisting deformation of the wings, leading to significant FSI effects

[48]. Full-process two-way FSI analysis of meter-scale flapping-wing takeoff remains comparatively underexplored, particularly for frameworks that couple flexible-wing deformation, unsteady flow, body motion, and complete trajectory evolution within one analysis.

Lack of Experimental Validation Data: The absence of sufficient experimental data required for model validation casts doubt on the credibility of simulation results

[49], [50]. This lack of reliable validation data severely restricts the practical application of FSI simulation in flapping-wing design.

The application of the Lattice Boltzmann Method (LBM) is the cornerstone of this study on fluid-solid interaction simulations. Its direct connection to the kinetic theory of gases allows for a deeper understanding and more detailed modeling of physical phenomena

[51]. The core principle of its algorithm involves dividing particle motion in physical space into two processes, namely collision and streaming:

$$f_{\alpha}^{*}(r,t)=f_{\alpha}(r,t)-\frac{1}{\tau}[f_{\alpha}(r,t)-f_{\alpha}^{eq}(r,t)]+\delta_{t}F_{\alpha}(r,t)$$

$$f_{\alpha}(r+e_{\alpha}\delta_{t},t+\delta_{t})=f_{\alpha}^{*}(r,t)$$

Where a particle collides with another particle at a node, and after one time step, it moves precisely from that node to an adjacent node. The collision process is local, and the streaming process is linear.

In this study, to achieve better computational accuracy and surface capturing capability, we employ the D3Q27 discrete velocity model with an octree structure, as shown in Fig. 2D. This model provides a higher number of velocity directions and thus provides a higher order scheme despite a slightly higher computational cost. The transformation matrix is:

The octree structure allows for the use of non-uniform lattice grids, adopting a hierarchical arrangement where the grid scale at each level is half that of the previous level. The turbulence modeling approach is Large Eddy Simulation (LES). This scheme introduces an additional turbulent eddy viscosity $\nu$ to model subgrid-scale turbulence:

$$\nu_{t}=\Delta_{f}^{2}\frac{\left(G_{\alpha\beta}^{d}G_{\alpha\beta}^{d}\right)^{\frac{3}{2}}}{\left(S_{\alpha\beta}S_{\alpha\beta}\right)^{\frac{5}{2}}+\left(G_{\alpha\beta}^{d}G_{\alpha\beta}^{d}\right)^{\frac{5}{4}}}$$

$$S_{\alpha\beta}=\frac{g_{\alpha\beta}+g_{\beta\alpha}}{2}$$

$$G_{\alpha\beta}^{d}=\frac{1}{2}\left(g_{\alpha\beta}^{2}+g_{\beta\alpha}^{2}\right)-\frac{1}{3}\delta_{\alpha\beta}g_{\gamma\gamma}^{2}$$

$$g_{\alpha\beta}=\frac{\partial u_{\alpha}}{\partial x_{\beta}}$$

Where$\Delta_{f}=C_{\omega}\Delta x$ is the filter scale, $S$ is the strain rate tensor of the resolved scales, and the constant $C_{\omega}$ is typically 0.325.

The near-wall region and boundary layer are simulated using wall functions. This enhanced approach incorporates the effects of both adverse and favorable pressure gradients:

$$\frac{U}{u_{c}}=\frac{\tau_{\omega}}{\rho u_{\tau}^{2}}\frac{u_{\tau}}{u_{c}}f_{1}(y^{+}\frac{u_{\tau}}{u_{c}})+\frac{dp_{\omega}/dx}{\left|dp_{\omega}/dx\right|}\frac{u_{p}}{u_{c}}f_{2}(y^{+}\frac{u_{p}}{u_{c}})$$

$$y^{+}=\frac{u_{c}y}{\nu}$$

$$u_{c}=u_{\tau}+u_{p}$$

$$u_{\tau}=\sqrt{\left|\tau_{w}\right|/\rho}$$

$$u_{p}=\left(\frac{\nu}{\rho}\left|\frac{dp_{\omega}}{dx}\right|\right)^{\frac{1}{3}}$$

$y$ is the normal distance from the wall, $x$ is the local ow direction tangentially to the wall, $u_{\tau}$ is the skin friction velocity, $\tau_{\omega}$ is the turbulent wall shear stress, $dp_{\omega}/dx$is the wall pressure gradient, $u_{p}$ is a characteristic velocity of the adverse wall pressure gradient and $U$ is the mean velocity at a given distance from the wall.

To achieve stable computational accuracy while ensuring computational resource consumption remains within acceptable limits, we conducted a grid independence verification. Using the 3 Hz flapping frequency benchmark case, simulations were performed with grid refinement sizes of 0.025 m, 0.0125 m, 0.00625 m, and 0.003125 m in the near-wall regions and wake zones. As shown in Table S1, when the grid refinement size was 0.00625 m, the relative computational error was -1.9% and -3.0%. This refinement level achieved high computational accuracy while maintaining acceptable computational time. Consequently, all simulations in this study adopted a refinement size of 0.00625 m.

**Table S1. Grid Independence Verification**

| **Grid refinement size [m]** | 0.025 | | 0.0125 | | 0.00625 | | 0.003125 | |
| --- | --- | --- | --- | --- | --- | --- | --- | --- |
| **Force components (Fx/Fy) [N]** | 4.44/3.06 | | 6.06/3.13 | | 6.84/2.85 | | 6.97/2.94 | |
| **Relative error [%]** |  | -29.2/-11.3 | | -11.4/9.8 | | -1.9/-3.0 | |  |
| **Computational cost [core·h/s]** | 735 | | 1260 | | 3480 | | 8064 | |

***B. Validation of the FSI method***

Validation of the FSI method was conducted through wind-tunnel deformation and force measurements. DIC measurements evaluate the accuracy of structural-deformation prediction, and lift/thrust measurements evaluate the accuracy of aerodynamic-force prediction. Together, they assess the two coupled outputs that govern the takeoff simulation.

Experiments were performed in a closed-circuit wind tunnel (Fig. S1B) with a test section of 6 m × 3.6 m. The flow quality was well controlled, with velocity instability, flow non-uniformity, and turbulence intensity all below 1%, and a streamwise static pressure gradient below 0.01/m. A total of eight test conditions were examined, corresponding to freestream velocities of 4 m/s, 8 m/s, and 12 m/s, combined with angles of attack of 0° and 10°. The flapping frequency was fixed at 3 Hz, with a stroke range of −5° to 40°.

Structural deformation was measured using digital image correlation (DIC) (Fig. S1A). Four representative points were selected: the mid-span and tip of the leading-edge spar, and the mid and outer regions of the trailing edge. The leading-edge points, characterized by higher stiffness, dominate the phase response of flapping motion, while the trailing-edge points capture large deformation induced by aeroelastic effects.

The DIC results show strong agreement between predicted and measured structural deformation across the tested conditions. Even at 12 m/s, 10° angle of attack, and point P3, the correlation coefficient is r = 0.9654 and NRMSE = 0.0947. The discrepancy increases gradually with freestream speed and angle of attack. Higher aerodynamic loading introduces small load-dependent variations in the experimental stroke speed relative to the prescribed sinusoidal kinematics, which contributes to the measured deformation difference.

**Fig. S1. Comparison between wind tunnel DIC measurements and FSI predictions.**

A. Locations of DIC measurement points.

B. Wind tunnel experimental setup.

C. Comparison of structural deformation at representative points, with corresponding correlation metrics between experiments and FSI predictions.

Figure S2 compares predicted and measured lift and thrust. At 4 m/s, the predicted force histories closely follow the measurements. As freestream speed and angle of attack increase, the phase-resolved difference grows. At 0° angle of attack, simulated lift becomes slightly lower than measured lift with increasing speed; at 10°, the lift difference shifts toward a modest overprediction at 8–12 m/s. Simulated thrust remains slightly lower than the measured history across the higher-speed cases, and its magnitude approaches zero as the propulsive margin decreases.

Three compatible effects explain the higher-load discrepancy. Increased aerodynamic resistance produces load-dependent variations in experimental flapping speed: the downstroke lengthens and the upstroke shortens relative to the prescribed sinusoidal kinematics, a pattern also reflected in the DIC comparison. Model idealization and localized grid-refinement resolution contribute additional phase and magnitude differences at higher load. As thrust approaches zero, a similar absolute difference produces a larger relative error.

The force-error trends also explain the direction of trajectory-level deviations. For states close to 0° angle of attack, underprediction of lift and thrust produces lower predicted vertical and forward acceleration than the measurements, leading to lower altitude and speed and a slower pitch recovery. For states near 10° angle of attack, the modest lift overprediction increases predicted vertical acceleration and the nose-up tendency, whereas thrust underprediction continues to reduce forward acceleration. The corresponding simulation-flight differences in altitude, speed, and pitch evolution follow these component-wise directions, providing a system-level consistency check. The zero-freestream, 3 Hz force comparison provides component-level validation relevant to the initial low-speed takeoff stage, and the 4–12 m/s DIC comparison supports deformation trends over the later higher-speed states.

**Fig. S2. Comparison of aerodynamic forces between wind tunnel measurements and FSI predictions.**

*C. Flight control system design*

To meet the requirements for lightweight design and high-precision control, the flight controller Decider-V1 developed in this study employs a microprocessor as its core processing unit. This controller collects signals from various sensors, executes algorithms for fused perception and navigation, trajectory planning, and flight control, then generates control commands for the flapping-wing drive motor and tail adjustment motor. During experiments, to avoid adverse effects of tail oscillation during low-speed phases and maintain consistency with simulation conditions, the flapping frequency was set constant at 5 Hz, while tail pitch angle followed predefined open-loop control rules.

The hardware system centers around the main controller, STM32F405RGT6, which serves as the central processing unit coordinating all modules. The power management module converts lithium battery voltage to provide stable power for components with varying input requirements. A remote-control link comprising a receiver, data transmission module, and ground station facilitates operator command transmission. Additional components include a flight log recorder, status indicator lights, and buzzers for flight data recording and controller status indication during debugging.

Hardware integration requires comprehensive consideration of sensor characteristics, dimensions, and mounting positions. The circuitry is divided into the main controller board and external modules (Fig. S3). The main board integrates the microcontroller minimum system, power management, power measurement, MEMS devices for pose measurement, and status display modules, with reserved interfaces for external components. Component layout design minimizes electromagnetic interference through crosstalk suppression while ensuring convenient module connection.

**Fig. S3. Flight Control PCB Design**

A. Main control board PCB design

B. External modules PCB design

C. Main control board layout (Top Layer)

D. External modules layout (Top Layer)

E. Main control board layout (Bottom Layer)

F. External modules layout (Bottom Layer)

Decider-V1's software architecture is built on FreeRTOS, an open-source real-time operating system (RTOS) offering three key advantages: First, its lightweight nature ensures high compatibility with the STM32F405RGT6 microcontroller. Second, its comprehensive features—including task management, semaphores, message queues, and software timers—enable efficient task scheduling. Third, the FreeRTOS-based programming framework supports modular and layered development, significantly enhancing debugging efficiency and future maintenance/upgrade flexibility.

***D. Wing-stiffness characterization***

Finite-element simulations were performed using an assembled-wing model consistent with the geometry, material configuration, and bonded interfaces of the manufactured wing. Eighteen representative points across six rib stations were used to obtain the 18 × 18 normal-compliance matrix and corresponding stiffness matrix under common boundary conditions. The complete numerical matrices and associated point and load definitions are provided in Supplementary File S2, and the regional stiffness values are summarized in Table S2 and Fig. S4B.

**Table S2. Regional equivalent rotational stiffness derived from the 18-point normal-compliance matrix.**

| **Rib station** | **K_theta(F-M) (N m/rad)** | **K_theta(M-R) (N m/rad)** | **F-M / M-R** |
| --- | --- | --- | --- |
| R1 | 15.1575 | 3.00636 | 5.0418 |
| R2 | 9.21784 | 3.03171 | 3.0405 |
| R3 | 4.24695 | 1.94994 | 2.1780 |
| R4 | 2.45644 | 1.27871 | 1.9210 |
| R5 | 1.98418 | 1.02191 | 1.9416 |
| R6 | 1.92134 | 0.987480 | 1.9457 |

The complete assembled-wing three-dimensional geometry is supplied as Supplementary File S1 in STEP (.stp) format. Figure S4A shows the wing structural layout, sampled rib stations, and compared regions.

Fig. S4. Wing structural model and regional stiffness distribution.

A. Planform representation of the assembled wing, sampled rib stations, and compared structural regions. F, M, and R denote points on the main spar, rear spar, and trailing edge, respectively.

B. Normalized equivalent rotational stiffness of the main-spar-to-rear-spar region and the region aft of the rear spar at six rib stations.
